# New Method for *Lawsonia intracelullaris* Quantification Based on Optical Density by Spectrophotometry

**DOI:** 10.3390/microorganisms13030568

**Published:** 2025-03-03

**Authors:** Mirtha E. Suarez-Duarte, Ricardo P. Laub, Renato L. Santos, Carlos E. R. Pereira, Talita P. Resende, Matheus D. Araujo, Paula A. Correia, Jessica C. R. Barbosa, Roberto M. C. Guedes

**Affiliations:** 1 Department of Clinic and Surgery, Veterinary School, Universidade Federal de Minas Gerais, Belo Horizonte 31270, Minas Gerais, Brazil; mirthasuarez009@hotmail.com (M.E.S.-D.); ricardolaub@gmail.com (R.P.L.); mtdaraujo@gmail.com (M.D.A.); medvetpaulacorreia@gmail.com (P.A.C.); carolballet12@hotmail.com (J.C.R.B.); 2 Department of Veterinary, Universidade Federal de Viçosa, Belo Horizonte 31270, Minas Gerais, Brazil; carlos.pereira@ufv.br; 3 Department of Animal Science, College of Food, Agriculture and Environmental Sciences, Ohio State University, Columbus, OH 43210, USA; resende.2@osu.edu

**Keywords:** optical density, proliferative enteropathy, indirect counting, RT-qPCR, spectrophotometer

## Abstract

Studies investigating the pathogenesis of *Lawsonia intracellularis* often require bacterial quantification in suspension. However, due to the organism’s fastidious growth requirements—being both intracellular and microaerophilic—traditional quantification methods, such as colony-forming unit counting, are not feasible. Currently, the only widely available method for quantifying *L. intracellularis* is real-time quantitative PCR (RT-qPCR). Unfortunately, the time required to perform RT-qPCR is incompatible with the bacterium’s limited survival outside its intracellular and microaerophilic environment. As a result, bacterial suspensions are typically quantified subjectively, based on the researcher’s experience for immediate use, with RT-qPCR conducted afterward. Optical density (OD) spectrophotometry is a rapid, although indirect, method of estimating bacterial concentrations in suspension, and it has been applied successfully to fast-growing prokaryotic species. Therefore, the objective of this study was to determine the correlation between RT-qPCR results and the optical density of *L. intracellularis* suspensions, with the goal of enabling the use of spectrophotometry for immediate bacterial quantification in experimental settings. Optical densities (ODs) were measured at 405 nm and 450 nm, using either a cuvette or microplate, while RT-qPCR was employed to establish a standard curve from samples of known concentration and to quantify the concentration of *L. intracellularis* in the test suspensions. Four comparison variations between OD and RT-qPCR were evaluated: (1) spectrophotometry at 405 nm using a cuvette vs. RT-qPCR; (2) spectrophotometry at 405 nm using a microplate vs. RT-qPCR; (3) spectrophotometry at 450 nm using a cuvette vs. RT-qPCR; and (4) spectrophotometry at 450 nm using a microplate vs. RT-qPCR. The tests were conducted in two independent replications, with each sample analyzed in duplicate. In all variations, the correlation between the bacterial concentrations determined by RT-qPCR and those estimated by OD was greater than 80%, with a statistical significance of *p* < 0.05. The following OD conversion equations for determining the number of microorganisms/mL were obtained: (1) f(x) = −7.438 × 10^8^ + 1.797 × 10^10^. x; (2) f(x) = 3.255 × 10^8^ + 3.003 × 10^9^. x; (3) f(x) = −8.006 × 10^8^ + 2.169 × 10^10^. x; (4) f(x) = 3.107 × 10^8^ + 3.758 × 10^9^. x. Here, “X” is the Ct value obtained by RT-qPCR. These findings enable researchers to improve the accuracy of their *L. intracellularis* experiments by utilizing optical spectrometry—a straightforward method that provides immediate results for determining bacterial concentration in suspensions.

## 1. Introduction

*Lawsonia intracellularis* is the etiological agent of proliferative enteropathy (PE), a diarrheal disease characterized by the thickening of the intestinal mucosa due to hyperplasia of intestinal epithelial cells affecting several animal species, mainly pigs and horses [1,2]. The first report of PE shows the presence of intracellular bacteria in association with the proliferation of the lining cells of the intestinal crypts [1]. Different names were attributed to the organism over time such as the *Campylobacter like-organism*, *Ileal symbiont intracellularis*, and *Ileobacter intracellularis* [2].

The onset of clinical signs is most commonly observed in post-weaning pigs, between 8 and 20 weeks of age, with diarrhea and decreased weight gain being the most characteristic clinical signs. Diarrhea occurs when there are significant intestinal lesions and may be presented clinically in acute and chronic forms. The acute form causes hemorrhagic diarrhea and/or sudden death in finishing pigs and breeding gilts. The chronic form mainly causes decreased daily weight gain and pasty diarrhea in growing animals [2].

The study of the pathogenesis of microorganisms is essential to understand clinical manifestations, as well as for the development of effective and modern strategies for controlling and preventing infections and for developing treatment protocols. Understanding pathogenesis, therefore, depends extensively on controlled studies, first performed in vitro and subsequently applied in vivo. The pathogenesis of *Lawsonia intracellularis* still needs clarification.

The isolation of *L. intracellularis* was successful in 1993 [1]. For the in vitro cultivation of *L. intracellularis*, a monolayer of cells permissive to infection is required, such as rat intestinal epithelial cells (IEC-15) (rat intestinal epithelium), McCoy (mouse fibroblasts), IPEC-J2 (porcine jejunum cell), INT-407 (human fetal intestine), CRL 1677 (rat colonic adenocarcinoma), and Caco-2 (human colorectal adenocarcinoma) [1,3,4], in a microaerophilic atmosphere with 82.2% nitrogen, 8.8% carbon dioxide, and 8% oxygen at 37 °C, which resembles the conditions of the intestinal environment [1].

To create this favorable environment, an alternative method for the cultivation of *L. intracellularis* was developed, where a gas mixture containing hydrogen, carbon dioxide, and nitrogen was placed in plastic bags used for vacuum storage of clothes. The flexibility of this method allowed the cultivation of the bacteria without the need for anaerobic jars or tri-gas ovens [5]. *L. intracellularis* causes a relevant intestinal disease in pigs and horses, as well as several other mammals, such as rabbits, called proliferative enteropathy, popularly known as ileitis [2].

It has been shown that *L. intracellularis* infects intestinal epithelial cells, especially in the crypts, leading to hyperplasia [1]. However, the details of this process have yet to be elucidated. After intestinal infection by experimental inoculation, intracellular bacteria can be visualized in intestinal epithelial cells and feces for one to three weeks, with the peak of infection and lesions three weeks after the challenge. The proliferative lesion and fecal excretion of the bacteria persist for approximately four weeks, but in some pigs, excretion can persist for 10 weeks [6].

There is little evidence of the dissemination of *L. intracellularis* beyond the intestinal epithelium or the presence of a significant inflammatory response in the intestine [7], unlike what is observed for other enteroinvasive agents such as *Salmonella* sp., *Shigella*, and *Listeria monocytogenes*. The specific bacterial determinants that confer pathogenicity and cause these distinct pathological effects are still poorly understood, but bacterial adhesion and entry occur through the apical surface of immature epithelial cells, in a process that appears to require a specific bacterial ligand–receptor interaction [8,9].

It is known that *L. intracellularis* is already present in mature enterocytes at the apex of villi 12 h after oral inoculation in pigs. It has also been demonstrated that the bacterium initially infects a variety of cell types in the crypts and villi, although it appears to be a preference for cells at the crypt-villus junction [10].

Furthermore, due to its intracellular characteristic, much of the applied research related to *L. intracellularis* is necessarily developed in vitro, such as studies to determine the minimum inhibitory concentration (MIC) [11] and epidemiology studies about how the fecal–oral transmission of *L. intracellularis* between mice and pigs [12] and, therefore, a quantification method is essential for the development of an accurate experimental design.

There are currently several research laboratories around the world working on investigating the pathogenesis of *L. intracellularis* in vitro. Unfortunately, all these laboratories experience difficulties when designing experiments that require the use of *L. intracellularis* at a predetermined concentration. Currently, real-time quantitative PCR (RT-qPCR) is the most used method for determining bacterial concentration in experimental inoculum [13]. However, even though the execution of an RT-qPCR is considered quick, it is long enough to be incompatible with maintaining the viability of *L. intracellularis* until the results are released. Therefore, the experiment is normally performed with a subjectively determined concentration and, only after completion of the RT-qPCR, is the real number of bacteria per unit volume defined.

Another technique for quantifying *L. intracellularis* is based on immunocytochemistry, an indirect immunoperoxidase stain using a monoclonal or polyclonal antibody specific to *L. intracellularis*. In this technique, serial concentrations are made from a pure bacterial suspension and then bacterial microorganisms are individually counted under an optical microscope [3]. However, like the RT-qPCR, this method does not offer an immediate result, in addition to requiring the availability of anti *L. intracellularis* antibody, which is not widely commercially available [14].

There are several methods for evaluating microbial concentration, such as measuring the number of cells, mass, or constituents. The most common techniques to determine bacterial concentration are viable plate counts, which measure the colony forming unit per milliliter (CFU/mL), and spectrophotometry, which determines the optical density (OD) of a liquid sample. The approach that measures the CFU/mL unit is highly sensitive; however, it is only feasible for live and culturable bacterial cells that generate visible colonies [15], which is not the case with *L. intracellularis*.

Optical density (OD) is an indirect measure of bacterial concentration, based on the turbidity of the inoculum. The number of bacteria in a suspension is directly proportional to the turbidity of the culture. Therefore, diluting a suspension to a specific OD corresponds to a known microbial concentration. This method is widely used in research laboratories because it provides a quick and simple way to estimate bacterial concentrations. By adjusting suspensions based on OD, researchers can rapidly achieve an estimated concentration, which can later be confirmed through more precise cell counting methods [4].

However, some factors are important for converting the OD to the number of microorganisms per volume unit, with the size of the microorganism being one of the most relevant. When a beam of monochromatic light passes through a spectrophotometer cuvette, some of the light undergoes refraction, reflection, absorption by reagents, and other undesirable interactions. To eliminate such interference, the instrument must be zeroed with a solution called a blank. The blank must contain all the constituents of the system, except the sample to be studied. For each set of determinations, as well as after changing the wavelength, the instrument must always be zeroed and calibrated with the tube containing the blank [4]. The spectrophotometer is a relatively easily accessible piece of equipment for most laboratories, but to date, there are no records of the standardization of a technique for quantifying *L. intracellularis* by optical density, as is already carried out for other bacteria.

*L. intracellularis* is a Gram-negative bacterium with a curved or sigmoid shape, 1.25 to 1.75 µm long and 0.25 to 0.43 µm wide, a cell wall with an external trilaminar envelope, and a single, mobile flagellum [7]. Its genome contains multiple VNTR (variable number of tandem repeats) sequences, which are important for identifying different strains of the bacteria isolated from field samples [16]. The circular genome of *L. intracellularis* has 1,719,014 base pairs (bp), consisting of a chromosome (1,457,619 bp) and three plasmids (plasmid A: 27,048 bp; plasmid B: 39,794 bp; and plasmid C: 194,553 bp) [17]. The size of *L. intracellularis* is unique in relation to other more widely studied intestinal bacteria, such as *E. coli*, with 1.2 to 2.0 µm in length by 0.5 µm in width [18], and *Salmonella*, with 2.0 to 5.0 µm in length and 0.7 to 1.5 µm in width [19].

These singularities in cell size make it difficult to use results from previous studies with other bacterial species to determine *L. intracellularis* concentration. Due to the need to have an immediate result quantification method for *L. intracellularis*, this work aimed to develop an optical density spectrophotometry (OD) method correlated to RT-qPCR for the quantification of *L. intracellularis* to increase the accuracy of the result and thus be able to use the rapid technique for quantifying *L. intracellularis* in laboratories in the future.

## 2. Materials and Methods

### 2.1. In Vitro Propagation of Lawsonia Intracellularis

The *L. intracellularis* vaccine strain (ATCC PTA-3457) was propagated in mouse fibroblasts (McCoy Cells; ATCC CRL-1696) as described previously [20]. In summary, McCoy cells were seeded in T-175 cm^3^ flasks and cultured in Dulbecco’s Modified Eagle Medium (DMEM; Gibco Invitrogen Corporation, New York, NY, USA) supplemented with 7% fetal bovine serum (FBS; Gibco Invitrogen Corporation, New York, NY, USA) and L-Glutamine (Gibco Invitrogen Corporation; New York, NY, USA) in a CO_2_ incubator, at 37 °C, for 24 h. When the cell monolayer reached 30% confluence, they were infected with *L. intracellularis* and then incubated at 37 °C in a controlled atmosphere with a mixture of gasses (10% O_2_, 10% CO_2_, and 80% nitrogen). Bacteria growth was evaluated for seven days until the cell monolayer reached a 100% confluence of highly infected cells [5,6]. Then, the supernatant was harvested from the flasks and concentrated centrifugation (4000× *g* for 20 min), and the cell monolayer was immersed in 0.1% potassium chloride, mechanically ruptured, and centrifuged in low speed to clean cell debris. The supernatant from this tube was saved and concentrated by centrifugation (4000× *g* for 20 min). Both supernatants, from the flasks and from the KCl lysis monolayer, were suspended in PBS and combined. This final bacteria suspension was filtered through a 0.80 µm filter to remove the remained cell debris. The resulting suspension was filtered through a 0.80 µm filter to remove cell debris. One aliquot of the suspension was then used to measure the optical density, and another aliquot was used for RT-qPCR. A negative control sample was based on mechanically lysed, non-infected McCoy cells, filtered through a 0.08 µm filter, to account for the turbidity caused by cell residues.

### 2.2. Spectrophotometric Analysis of Optical Density (OD) of L. intracellularis Culture

The OD of the culture was determined based on the expression OD = log (I0/I), where I0 is the intensity of the incident light, and I is the intensity of the light transmitted through the suspension of bacteria. Two wavelengths were tested: 405 and 450 nm. Samples from the *L. intracellularis* culture were placed in 15 mL conical tubes in serial concentrations. The first suspension corresponded to resuspended, purified, and filtered material coming directly from the cell lysate. From this initial sample, subsequent concentrations were made, in a 2:1 ratio, with a total of five concentrations s being made (D1, D2, D3, D4, D5). The number of concentrations was determined based on a pilot test, in which the OD of higher dilutions was not detected by the spectrophotometer.

To read the optical density, plastic cuvettes and 96-well microplates were used. The plastic cuvettes were 4.40 cm high and 1.25 cm long and were filled with 1 mL of the bacterial suspension. The 96-well microplates (Sarstedt; Numbrecht, Germany) were translucent with external well dimensions of 85.4 × 127.6 × 3 × 14.40 mm and were filled with 200 µL of bacterial suspension. The equipment used for spectrophotometry in the cuvettes was the Bio-Rad Smart Spec 3000 (Bio-Rad, Sao Paulo, SP, Brazil), and for measurement on microplates, the semi-automatic Elisa reader MCL-2100C (Agilent; Santa Clara, CA, USA) was used.

### 2.3. Real Time Quantitative PCR

To perform the real-time PCR and its subsequent comparison with the optical density results, aliquots of the *L. intracellularis* suspension dilutions used in spectrometry analyses were used for DNA extraction with the DNeasy commercial extraction kit (Blood & Tissue Kit; Qiagen Inc., Toronto, ON, Canada), according to the manufacturer’s instructions. For the RT-qPCR, the primers bcL.intra114f (5′CACTTGCAAACAATAAACTTGGTCTTC-3′) and bcL.intra-263r (5′CATTCATATTTGTACTTGTCCCTGCA-3′) were used, associated with the probe intra201p (TCCTTGATCAATTTGTTGTGGATTGTATTCAAGG), which targets the aspartate ammonia-lyase gene (*aspA*), with a detection limit between bases 10^4^–10^11^. The reaction was performed in a final volume of 20 μL, consisting of 20 μL primer and probes for the TaqMan system and a final concentrations of 400 nM for each primer and 80 nM for the TaqMan probe and PCR Mastermix (TaqMan Universal PCR Mastermix; Applied Biosystems; Sao Paulo, SP, Brazil), according to the manufacturer’s instructions. Samples were placed in 96-well plates and amplified in a thermocycler ViiATM 7 Real-Time PCR System (Applied Biosystems), with the following amplification conditions: 2 min at 50 °C, 10 min at 95 °C, 40 cycles of 15 s at 95 °C, and 1 min at 60 °C. All reactions were performed in duplicate, and each reaction included the standard curve and negative control. Results were analyzed using QuantStudioTM software RealTime PCR v1.2. The standard curve was outlined in accordance with other RT-qPCR studies [21].

### 2.4. Statistical Analysis

Results of RT-qPCR and spectrophotometry were used to determine the correlation between OD and Ct, with subsequent regression analysis to determine the equation for transforming OD values into the number of bacteria per mL. Based on the variation in wavelengths for spectrophotometry and the container used to read the samples (cuvette or 96-well microplates), four experimental groups were created: (1) spectrophotometry at 405 nm using cuvette vs. RT-qPCR; (2) spectrophotometry at 405 nm on microplate vs. RT-qPCR; (3) spectrophotometry at 450 nm in cuvette vs. RT-qPCR; and (4) spectrophotometry at 450 nm using microplate vs. RT-qPCR. For each experimental group, two independent tests were carried out, with each sample analyzed in duplicates. To verify the repeatability of the results, two independent tests were designed (test 1 and test 2). Statistical analysis was performed using the R software (R Core Team, version 4.4.3) [22].

## 3. Results

### 3.1. Spectrophotometry and RT-qPCR

The data obtained for the optical spectrophotometry and RT-qPCR for test 1 and test 2 are in Table 1 and Table 2, respectively. As expected, serial dilutions of the bacterial suspensions were accompanied by an increase in RT-qPCR Ct values and a decrease in OD (Table 1 and Table 2).

### 3.2. Regression Analysis

The correlation results between the OD obtained by spectrophotometry and the quantity of bacteria determined by converting the Ct obtained in RT-qPCR using the standard curve are presented in Table 3. The correlation analysis demonstrated that regardless of the wavelength and type of container used to analyze the sample in spectrophotometry (cuvette or microplate), the correlation with the RT-qPCR results was considered high, with R^2^ values > 0 and 8 for all tested variations, and a significance level of *p* < 0.05 for all of them.

From this analysis, the equation for converting OD to number of bacteria per mL was determined, so that each of the four combinations between wavelength and container for spectrophotometry can be used according to the availability in each laboratory calculations of amount of *L. intracellularis* per mL of bacterial suspension based on the Ct values obtained by the RT-qPCR reaction are in Table 4.

### 3.3. Transformation of OD to mL of Bacterial Suspension

For the realization of the optical density (OD), optical spectrophotometry transformed the number of *L. intracellularis* per mL, for both test 1 and test 2, respectively, as shown in Table 5. In these tables, we can see the OD results transformed into a logarithmic basis, which finally allows us to immediately quantify *L. intracellularis* in the suspension. Table 4 and Table 5 contain the ODs with the respective quantification of bacteria.

## 4. Discussion

Due to its fastidious type of in vitro cultivation in permissive eukaryotic cells and in a specific microaerophilic atmosphere, *L. intracellularis* cannot be quantified by traditional methods commonly utilized for other bacterial species, such as CFU counting, for example. Other quantification techniques such as flow cytometry and optical microscopy are sensitive, precise, and have the ability to distinguish between live and dead bacteria when the appropriate dyes are used [23].

However, these methods have some limitations. In addition to both requiring a relatively long time to be performed, flow cytometry requires high-cost equipment, and optical microscopy is only effective when there is easy visualization of the bacterial agent, which is not the case with *L. intracellularis*. Immunocytochemistry and RT-qPCR have been used to quantify *L. intracellularis* in suspensions. However, as these techniques do not provide an immediate result, the researcher ends up using a subjective assessment of bacterial concentrations, which is only confirmed or rectified after the technique has been completed.

Optical spectrophotometry is a technique used to determine the amount of a substance or elements in solution or suspension in a sample. Performing OD measurements is not complex, particularly when performed on plate or cuvette readers, as these measurements are extremely fast, cheap, simple, relatively seamless, have high throughput, and are readily automated [24]. The spectrophotometer is a laboratory equipment frequently present in most research institutes. Due to these characteristics, optical spectrophotometry is a quick, although indirect, method of estimating the concentrations of bacteria suspended in liquid media [25].

Despite the accessibility of a spectrophotometer for most laboratories and its use for quantifying bacteria such as *E. coli*, *Salmonella*, and some viruses over a long period of time [26], to date, there are no records of the standardization of a technique for quantifying *L. intracellularis* by spectrophotometry/optical density.

With the equations for converting OD into number of bacteria per mL developed in a pioneering manner in this study, it becomes possible to quickly and serially quantify a culture of *L. intracellularis* in any laboratory that has a spectrophotometer available that can read wavelengths of 405 or 450 nm, whether for reading plates or cuvettes [27]. Considering that this is the first study on optical density of *L. intracellularis*, we decided to start with shorter wavelengths, but it is also very important to test other longer wavelengths such as an OD of 600 nm in future studies, remembering that each culture has a characteristic absorption spectrum, which means that it can absorb more or less radiation at certain wavelengths and that a longer wavelength can allow deeper penetration and cellular interaction [28,29].

By combining the quantitative calculation methodology of real-time polymerase chain reaction (RT-qPCR) with spectrophotometric reading, an indirect quantitative method for bacterial counting with immediate results is available.

While this method of study is already widely and routinely used for the contagion of extracellularly growing bacteria, it is the first study to determine the quantification of *L. intracellularis* in suspension using the optical density spectrophotometry technique, a fast, accessible, and easy-to-perform technique that will facilitate the next stages of pathogenesis studies and other necessary studies with this bacterium in laboratories around the world. The use of this technique will favor accuracy and repeatability for the development of experiments both in vitro and in vivo with *L. intracellularis*.

## 5. Conclusions

In this work, an immediate quantification method for *L. intracellularis* was developed for the first time using optical density spectrophotometry correlated with qPCR. This method is quick, easy, highly reproducible, and not laborious, and is therefore easy to implement as a quantification method into the routine of *L. intracellularis* cultivation by researchers working in vitro and in vivo in laboratories around the world. 

## Figures and Tables

**Table 1:** Optical density values for test 1, referring to *L. intracellularis* suspensions in serial concentrations of 2:1, from suspension D1.

Concentrations	405 nm Cuvette	450 nm Cuvette	405 nm Microplate	450 nm Microplate	RT-qPCR (Ct)
D1	0.604	0.487	0.161	0.137	15.706
D2	0.302	0.245	0.106	0.091	16.094
D3	0.149	0.125	0.085	0.073	16.520
D4	0.077	0.064	0.073	0.063	18.657
D5	0.026	0.024	0.066	0.057	19.191

**Table 2:** Optical density values for test 2, referring to *Lawsonia intracellularis* suspensions in serial concentrations of 2:1, from suspension D1.

**Concentrations**	**405 nm** **Cuvette**	**450 nm** **Cuvette**	**405 nm** **Microplate**	**450 nm** **Microplate**	**RT-qPCR (Ct)**
D1	0.381	0.305	0.178	0.152	16.708
D2	0.182	0.147	0.120	0.102	17.392
D3	0.076	0.057	0.086	0.075	18.282
D4	0.044	0.032	0.068	0.057	19.004
D5	0.027	0.019	0.058	0.051	20.361

**Table 3:** Correlation results between spectrophotometry and RT-qPCR, according to the evaluated parameters of wavelength and container.

Parameters	R^2^	Significance Level (*p*)	Equation
RT-qPCRq vs. OD on microplate at 405 nm	0.8149	0.0359	*f*(*x*) *=* −7.438 × 10^8^ + 1.797 × 10^10^. *X*
RT-qPCR vs. DO in cuvette at 405 nm	0.8423	0.0279	*f*(*x*) *=* 3.255 × 10^8^ + 3.003 × 10^9^. *X*
RT-qPCR vs. OD on microplate at 450 nm	0.82	0.0343	*f*(*x*) = −8.006 × 10^8^ + 2.169 × 10^10^. *X*
RT-qPCR vs. DO in cuvette at 450 nm	0.846	0.027	*f*(*x*) *=* 3.107 × 10^8^ + 3.758 × 10^9^. *X*

**Table 4:** Result of Ct transformation obtained by RT-qPCR for the amount of *L. intracellularis*/mL of tsest 1 and 2, for spectrophotometry at 450 nm and cuvette reading, and the equation of f(x) = −3.584x + 48.983.

**Test 1 Sample**	**Ct**	**Log (X = −Y − 48.983/3.584)**	***L. intracellularis*/mL**
D1	15.706	9.285	1.93 × 10^9^
D2	16.094	9.177	1.50 × 10^9^
D3	16.520	9.058	1.14 × 10^9^
D4	18.657	8.461	2.89 × 10^8^
D5	19.191	8.313	2.05 × 10^8^
**Test 2 Sample**	**Ct**	**Log (X = −Y − 48.983/3.584)**	***L. intracellularis*/mL**
D1	16.844	8.967	9.28 × 10^8^
D2	17.568	8.765	5.83 × 10^8^
D3	18.334	8.552	3.56 × 10^8^
D4	18.954	8.379	2.39 × 10^8^
D5	20.451	7.961	9.14 × 10^7^

**Table 5:** Result of optical density transformation (OD) for the amount of *Lawsonia intracellularis* per mL of tests 1 and 2 in a cuvette with spectrophotometric reading at 450 nm.

Test 1 Sample	OD	*L. intracellularis*/mL Cell Count (CC)	Test 2 Sample	OD	*L. intracellularis*/mL Cell Count (CC)
D1	0.305	1.46 × 10^9^	D1	0.487	2.14 × 10^9^
D2	0.15	8.63 × 10^8^	D2	0.25	1.23 × 10^9^
D3	0.06	5.25 × 10^8^	D3	0.13	7.80 × 10^8^
D4	0.03	4.31 × 10^8^	D4	0.06	5.51 × 10^8^
D5	0.02	3.82 × 10^8^	D5	0.02	4.01 × 10^8^

## Data Availability

The original contributions presented in this study are included in the article. Further inquiries can be directed to the corresponding author.
